# Impact of a Novel Anticoccidial Analogue on Systemic *Staphylococcus aureus* Infection in a Bioluminescent Mouse Model

**DOI:** 10.3390/antibiotics11010065

**Published:** 2022-01-06

**Authors:** Hang Thi Nguyen, Henrietta Venter, Lucy Woolford, Kelly Young, Adam McCluskey, Sanjay Garg, Stephen W. Page, Darren J. Trott, Abiodun David Ogunniyi

**Affiliations:** 1 Australian Centre for Antimicrobial Resistance Ecology, School of Animal and Veterinary Sciences, The University of Adelaide, Roseworthy, SA 5371, Australia; hang.t.nguyen@adelaide.edu.au; 2 Department of Pharmacology, Toxicology, Internal Medicine and Diagnostics, Faculty of Veterinary Medicine, Vietnam National University of Agriculture, Hanoi 100000, Vietnam; 3 Health and Biomedical Innovation, Clinical and Health Sciences, University of South Australia, Adelaide, SA 5000, Australia; rietie.venter@unisa.edu.au; 4 School of Animal and Veterinary Sciences, The University of Adelaide, Roseworthy Campus, Roseworthy, SA 5371, Australia; lucy.woolford@adelaide.edu.au; 5 Chemistry, School of Environmental & Life Sciences, University of Newcastle, Callaghan, NSW 2308, Australia; kelly.a.young@newcastle.edu.au (K.Y.); adam.mccluskey@newcastle.edu.au (A.M.); 6 Clinical and Health Sciences, University of South Australia, Adelaide, SA 5000, Australia; sanjay.garg@unisa.edu.au; 7 Neoculi Pty Ltd., Burwood, VIC 3125, Australia; swp@advet.com.au

**Keywords:** NCL179, colistin, robenidine, Gram-positive bacteria, *Staphylococcus aureus*, Gram-negative bacteria, multidrug-resistance, bioluminescence, antibiotic combination

## Abstract

In this study, we investigated the potential of an analogue of robenidine (NCL179) to expand its chemical diversity for the treatment of multidrug-resistant (MDR) bacterial infections. We show that NCL179 exhibits potent bactericidal activity, returning minimum inhibitory concentration/minimum bactericidal concentrations (MICs/MBCs) of 1–2 µg/mL against methicillin-resistant *Staphylococcus aureus*, MICs/MBCs of 1–2 µg/mL against methicillin-resistant *S. pseudintermedius* and MICs/MBCs of 2–4 µg/mL against vancomycin-resistant enterococci. NCL179 showed synergistic activity against clinical isolates and reference strains of *Acinetobacter baumannii*, *Escherichia coli*, *Klebsiella pneumoniae* and *Pseudomonas aeruginosa* in the presence of sub-inhibitory concentrations of colistin, whereas NCL179 alone had no activity. Mice given oral NCL179 at 10 mg/kg and 50 mg/kg (4 × doses, 4 h apart) showed no adverse clinical effects and no observable histological effects in any of the organs examined. In a bioluminescent *S. aureus* sepsis challenge model, mice that received four oral doses of NCL179 at 50 mg/kg at 4 h intervals exhibited significantly reduced bacterial loads, longer survival times and higher overall survival rates than the vehicle-only treated mice. These results support NCL179 as a valid candidate for further development to treat MDR bacterial infections as a stand-alone antibiotic or in combination with existing antibiotic classes.

## 1. Introduction

Extensive use of antibiotics for the treatment of bacterial infections in humans and animals, for food preservation and in agricultural industries over many decades has resulted in the emergence and increase of multidrug-resistant (MDR) bacteria [1,2]. In particular, MDR ESKAPE (*Enterococcus faecium, Staphylococcus aureus, Klebsiella pneumoniae, Acinetobacter baumannii, Pseudomonas aeruginosa* and *E. coli*/*Enterobacter* spp.) pathogens are associated with high mortality rates and increased health care setting costs [3,4,5,6]. It is estimated that antimicrobial resistance may cause 10 million deaths per year worldwide, exceeding the estimated combined deaths from cancers and diabetes and an investment of $100 trillion US dollars annually after 2050 unless adequately addressed [7].

It is becoming increasingly evident that the golden era of antibiotics for the treatment of MDR bacterial infections has lapsed [8], and the effectiveness of single and combination therapy against MDR ESKAPE pathogens is declining [8,9]. Many recommended antibiotics against ESKAPE pathogens have been withdrawn, and few antibiotics or antibiotic combinations have been added to the Clinical and Laboratory Standards Institute (CLSI) guidelines since 2010 [10]. Alarmingly, there is an increasing incidence of resistance reported against critically important antibiotics, such as the carbapenems: doripenem, imipenem, meropenem and ertapenem [10,11]. The antimicrobial resistance crisis has been worsened by Gram-negative bacterial (GNB) infections, particularly KAPE infections, as the GNB outer membrane often prevents antibiotics from gaining entry to their site of action [12]. There are several recently approved antibiotics, such as ceftazidime-avibactam, cefiderocol, imipenem + cilastatin + relebactam, and plazomicin, for the treatment of GNB infections [13,14,15,16,17]. However, these antibiotics are far from ideal therapeutic options to fully combat the emergence of MDR GNB infection [18]. Currently, polymyxins (such as polymyxin B (PMB) and colistin) are considered last-resort antibiotics for the treatment of MDR GNB infections particularly due to their specific targeting of the outer membrane [19,20,21]. Nonetheless, resistance to polymyxins is emerging via multiple mechanisms [19,20,22]. Moreover, many studies have reported that the use of polymyxins is associated with nephrotoxicity, neurotoxicity and neuromuscular blockade [23]. Consequently, it is imperative to find alternative options for the treatment of MDR bacterial infections, particularly those caused by GNB KAPE pathogens [18,24]. Given the current magnitude of MDR infections, international institutions such as the European Union, G20, UN General Assembly, World Bank, World Health Organization (WHO), and the USA and UK governments have issued documents calling for urgent action and development of new antibiotics with novel mechanisms of action against MDR ESKAPE pathogens [25,26].

**Figure 1:**
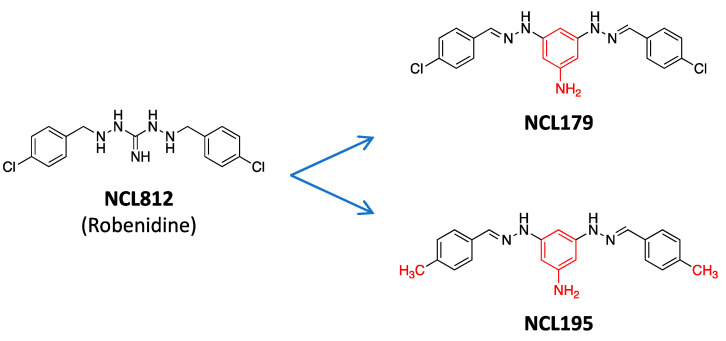
Chemical structures of NCL812 (Robenidine), NCL179 and NCL195. Red colouration highlights the structural changes in NCL179 and NCL195 relative to NCL812. The guanidine to triaminopyrimidine novel bioisosteric modification of NCL812 yielded NCL179 (2,2′-*bis*[(4-chlorophenyl)methylene] carbonimidic dihydrazide) and NCL195 (4,6-bis(2-((E)-4-methylbenzylidene)hydrazinyl)pyrimidin-2-amine). NCL179 contains a halogen (chlorine) that distinguishes it from NCL195.

To address the critical need for new antimicrobials, our team has developed structure–activity data associated with the anticoccidial robenidine (NCL812), used since the 1970s to control coccidiosis in commercial poultry and rabbit production. To date, we have generated > 270 analogues with selected members showing antimicrobial activity [27]. Among these, NCL195 and NCL179 are particularly promising pyrimidine analogues differing in the presence of a methyl group (CH_3_) at C4 of the terminal aromatic moieties of NCL195, while NCL179 conserved the original halogen (chlorine) of robenidine (Figure 1).

To date, we have performed several in vitro and in vivo studies that indicate NCL195 is a promising candidate for further pharmaceutical development for the treatment of MDR Gram-positive bacterial (GPB) infections and have shown its in vitro activity against MDR GNB in the presence of sub-inhibitory concentrations of different adjuvants [4,5,28,29]. In particular, the NCL195 + colistin combination has shown efficacy against both colistin-susceptible and -resistant *E. coli* in mouse models (manuscript in preparation). Given the close chemical relationship between NCL195 and NCL179, we postulated that NCL179 is likely to show in vitro and in vivo antimicrobial profiles similar to NCL195 but with potentially unique characteristics, thereby expanding the clinical utility of robenidine analogues. Thus, this study aims to evaluate the in vitro activity of NCL179 against human and animal MDR GPB pathogens, including methicillin-resistant *S. aureus* (MRSA), methicillin-resistant *S. pseudintermedius* (MRSP) and porcine vancomycin-resistant enterococci (VRE), as well as examine its activity against MDR GNB in the presence of sub-inhibitory concentrations of colistin. In addition, the efficacy of NCL179 against GPB was investigated in our validated bioluminescent mouse model of *S. aureus* sepsis.

## 2. Materials and Methods

### 2.1. Antimicrobials and Chemicals

NCL179, an analogue of NCL812, was synthesised at the University of Newcastle (Callaghan, NSW, Australia). Amikacin, daptomycin, kanamycin, tetracycline and colistin were purchased from Sigma-Aldrich (Australia). These antibiotics were prepared as 25.6 mg/mL stock solutions as follows: NCL179, tetracycline and daptomycin were dissolved in DMSO while amikacin and kanamycin were dissolved in water. All compounds were aliquoted in 1 mL quantities and stored at −20 °C in the dark. The NCL179 formulation used in efficacy experiments was prepared as a 50 mg/mL solution in a vehicle at Clinical and Health Sciences, University of South Australia, SA 5000, Adelaide, Australia. Moxifloxacin (drug control) was obtained as Avelox IV 400, 1.6 mg/mL (Bayer, Australia) or prepared in almond oil at 6 mg/kg (BovaVet, Caringbah, NSW, Australia).

### 2.2. Organisms and Growth Conditions

Clinical MRSP isolates from skin wounds, ears, abscessed joints and dog urine and clinical MRSA isolates were provided by the Australian Centre for Antimicrobial Resistance Ecology (ACARE), School of Animal and Veterinary Sciences, The University of Adelaide, Roseworthy, SA. Vancomycin-resistant enterococci (VRE) were obtained from the University of South Australia collection. Bioluminescent *S. aureus* ATCC 12600 (Xen29; PerkinElmer) was used for the efficacy testing of NCL179. Reference strains *S. aureus* ATCC 29213, *A. baumannii* NCIMB 12457, *A. baumannii* ATCC 19606, *E. coli* ATCC 35218, *E. coli* ATCC 25922, *E. faecalis* ATCC 29212, *K. pneumoniae* ATCC 13883, *K. pneumoniae* ATCC 33495 and *P. aeruginosa* ATCC 27853, PAO1 were obtained from SA Pathology, Adelaide, Australia. Bioluminescent *E. coli* (Xen14, a derivative of *E. coli* WS2572) and bioluminescent *P. aeruginosa* (Xen41, a derivative of strain PAO1) were purchased from PerkinElmer Inc (Waltham, MA, USA). Clinical human *A. baumannii* isolates (*n* = 16) were provided by South Australia Pathology; clinical human *E. coli* isolates (*n* = 20), clinical human *K. pneumoniae* isolates (20) and clinical human *P. aeruginosa* isolates (*n* = 22) were collected from Flinders Medical Centre (Bedford Park, SA, Australia). All bacteria were stored at −80 °C in Luria–Bertani (LB) broth containing 50% glycerol at the Microbiology laboratory, Level 4, Basil Hetzel building, University of South Australia, SA 5000, Adelaide, Australia.

All bacterial identities were confirmed by matrix-assisted laser desorption/ionisation time-of-flight mass spectroscopy (MALDI-TOF/MS) (microflex^®®^LT/SH Biotyper; Bruker Daltonik, Leipzig, Germany) at ACARE prior to antimicrobial susceptibility testing. Bacteria were grown overnight on horse blood agar (HBA) and in LB broth. *E. coli* Xen14 was grown on HBA containing 30 μg/mL kanamycin, *P. aeruginosa* Xen41 was grown on HBA containing 60 μg/mL tetracycline and *S. aureus* Xen29 was grown on HBA containing 200 μg/mL kanamycin. Colistin-resistant Xen14 were generated by daily serial subculture in increasing concentrations of colistin from 0.25 μg/mL to 256 μg/mL over 12–15 days as described previously [28].

### 2.3. Antimicrobial Susceptibility Testing

The minimum inhibitory concentrations (MIC) for NCL179, daptomycin and amikacin against clinical MRSP, MRSA, VRE and GNB isolates were determined in round-bottom 96-well microtitre plates (Sarstedt 82.1582.001, Nümbrecht, Germany), using the modified broth micro-dilution method recommended by the Clinical and Laboratory Standards Institute [30], as described previously [5]. Briefly, antimicrobial challenge plates were prepared by serial two-fold dilutions of 100× stock solutions (25.6 mg/mL) of NCL179 and daptomycin in DMSO, with the amikacin in water. Each dilution was then further diluted 1:100 in LB broth in 96-well plates. NCL179, daptomycin and amikacin concentrations ranged from 0.03 µg/mL to 256 µg/mL, and each MIC test was carried out in duplicate and performed on two separate occasions. Negative growth control was LB broth only; positive growth control was a bacterial suspension in LB broth. The MICs for NCL179 or daptomycin against Xen29 or *S. aureus* ATCC 29213 in the presence of 10% (vol/vol) foetal bovine serum (FBS) was also examined. The minimum bacterial concentration (MBC) was recorded as the lowest concentration of each test compound, at which a 99.9% colony count reduction was observed on the plate [31].

The interaction between NCL179 and colistin for anti-GNB activity was assessed by a modification of the standard checkerboard assay described previously [32,33]. The antibiotic challenge plates were prepared as described for the MIC testing above. One microliter of NCL179 solution from each combination was dispensed along the abscissa (from row A to F) of the 96-well microtiter plates; the second compound (colistin) was dispensed along the ordinate (from column 3 to column 12) using a 12.5 µL electronic multichannel pipette (VIAFLO Voyager II, Biotools) followed by the addition of 88 µL of LB broth. Thereafter, 10 µL of bacterial suspension was added to each well. One plate was used for each isolate, and the plates were incubated at 37 °C for 20 h and observed visually and by *A*_600nm_ measurements. The interaction of two antibiotics was calculated as the fractional inhibitory concentration index (FICI) described previously [32,33,34]. The dose reduction index (DRI) was used to describe the difference between the effective dose of NCL179 in combination with colistin from the individual dose of each compound [33]. A DRI is beneficial if it is higher than 1. An example of a checkerboard assay of NCL179 + colistin combination against *E. coli* ATCC 35218 is depicted in Figure S1 (Supplementary Materials).

### 2.4. Time-Dependent Growth Inhibitory Assay

The time- and concentration-dependent activity of NCL179 against one clinical MRSP isolate, one clinical MRSA isolate, one clinical VRE isolate, and against one reference *E. coli* Xen14, one colistin-resistant *E. coli* Xen14, one reference *A. baumannii* strain, one colistin-resistant *A. baumannii* clinical isolate, one reference *P. aeruginosa* strain and one reference *K. pneumoniae* strain in the presence of sub-inhibitory concentrations of colistin was determined in a kinetics assay by optical density (*A*_600nm_) measurements for 18 h on a Cytation 5 Multi-Mode Reader (BioTek, Winooski, VT, USA).

### 2.5. In Vitro Cytotoxicity Assays

We assayed NCL179 alone and in combination with colistin at 0.5 μg/mL for in vitro cytotoxicity using a panel of adherent mammalian cell lines: Hep G2 (human hepatocellular carcinoma cell line) and HEK293 (human embryonic kidney cell line) as described previously [28]. Briefly, cell lines were maintained in Dulbecco’s Modified Eagle’s Medium (DMEM; Gibco Cat No: 12430, Thermo Fisher Scientific, SA, Australia) supplemented with 10% FBS and 1% PenStrep (100 U/mL Penicillin and 100 μg/mL Streptomycin) at 37 °C, 5% CO_2_ and passaged every three days. Assays were performed in duplicates in black flat-bottom 96-well tissue culture trays (Eppendorf Cat No: 0030741013, Hamburg, Germany) seeded with ~1.5 × 10^4^ cells per well. After 24 h incubation, the media was removed, the cells were washed once with a medium without antibiotics, and the fresh medium supplemented with 10% (*v*/*v*) FBS was added. Then, either NCL179 alone or in combination with colistin was added to each well in doubling dilutions starting at the same concentrations used for MIC testing, using wells containing 1% DMSO only, colistin alone at 0.5 µg/mL and 64 µg/mL ampicillin as controls. The viability of each cell line in the presence of NCL179 alone, NCL179 + colistin combination or colistin alone (using 1% DMSO only and 64 μg/mL ampicillin as controls) was assessed at 1 h intervals for 20 h at 37 °C, 5% CO_2_ on a Cytation 5 Cell Imaging Multi-Mode Reader (BioTek, Winooski, VT, USA) using the RealTime-Glo TM MT Cell Viability Assay reagent (Promega, Madison, WI, USA).

### 2.6. Hemolysis Assay

The potential for NCL179 to lyse red blood cells (RBCs) was examined on fresh donor human RBCs using serial 2–fold dilutions of NCL179 (starting at 128 μg/mL) in quadruplicates in a round-bottom 96-well microtiter plate (Sarstedt 82.1582.001, Nümbrecht, Germany) according to the protocol described previously [28]. Serial 2–fold dilutions of ampicillin (starting at 128 μg/mL), 1% Triton X100 or PBS only served as controls. The experiment was carried out on two separate occasions using different fresh donor human RBCs. The hemolytic titer for NCL179 was determined as the reciprocal of the dilution at which 50% of erythrocytes were lysed as determined at *A*_450nm_.

### 2.7. Ethics Statements

Outbred 5 to 6-week-old male CD1 (Swiss) mice (weighing between 25 g to 30 g), obtained from the Laboratory Animal Services breeding facility of the University of Adelaide, were used to test the safety of NCL179 and assess its efficacy against *S. aureus*. The mice had access to food and water ad libitum. The Animal Ethics Committee of The University of Adelaide (approval number S-2015-151) reviewed and approved all animal experiments. The study was conducted in compliance with the Australian Code of Practice for the Care and Use of Animals for Scientific Purposes (8th Edition 2013) and the South Australian Animal Welfare Act 1985.

### 2.8. Safety Testing of NCL179 Following Oral Administration to Mice

In order to ascertain that two oral doses (8 h apart) or four oral doses (4 h apart) of either 10 mg/kg or 50 mg/kg NCL179 would be safe to administer to the mice, safety studies were conducted using oral administrations of 10 mg/kg or 50 mg/kg of NCL179 to three mice, using the vehicle (20% [*v*/*v*] DMSO in PEG400) as a control. The mice were observed for clinical signs (weight loss, dull/ruffled coat, poor posture, pale or sunken eyes, change in behaviour, dehydration, bleeding from the orifice, diarrhoea, breathing and reluctance to move) and the data recorded on a Clinical Record Sheet approved by the Animal Ethics Committee of The University of Adelaide. At the conclusion of the experiment, the mice were humanely killed, and sections of the heart, lung, liver, kidneys, spleen, stomach and small and large intestines were collected and subjected to histopathological examination.

### 2.9. Agar Well Diffusion Method

The NCL179 and moxifloxacin (drug control) formulations were tested for antibacterial activity using the agar well diffusion method to ensure that the drugs were released from the vehicle as a reference for the interpretation of in vivo activity in mice. For this assay, a few colonies of an overnight HBA culture of Xen29 were suspended in saline equivalent to 0.5 McFarland standard (*A*_600nm_ = 0.1). A sterile swab was then dipped in the 0.5 McFarland standard bacterial suspension and used to streak over the entire surface of a sterile plate count agar plate. Punch holes were then made on the agar plates using an 8 mm diameter biopsy punch (Livingstone International Pty Ltd., Sydney, NSW, Australia), and a 30 μL amount of the NCL179 formulation equivalent to a single treatment dose in mice was placed in the well. The antimicrobial activity of each drug was then determined by measuring and comparing the zone of inhibition with that of the vehicle only.

### 2.10. Histopathological Examination

Mouse organs (heart, lung, stomach, liver, spleen and small and large intestine) collected from the oral safety experiment were fixed in 10% neutral-buffered formalin (ChemSupply Australia Pty Ltd., Gillman, SA, Australia) and processed routinely, embedded in paraffin blocks and sectioned to a thickness of 4 µm. The hematoxylin and eosin-stained sections were observed and recorded under light microscopy. Photomicrographs were captured using a DP25 camera and LabSens software (Olympus, Tokyo, Japan).

### 2.11. Oral Efficacy Testing of NCL179 Following Systemic Challenge of Mice with Bioluminescent S. aureus

For efficacy testing experiments of NCLl79 against *S. aureus*, mouse-passaged Xen29 was used. Bacteria were grown in LB broth at 37 °C to *A*_600nm_ of 0.5 (equivalent to approximately 1.5 × 10^8^ CFU/mL). Three groups of mice (*n* = 6 mice per group) were challenged intraperitoneally (IP) with approximately 3 × 10^7^ CFU of Xen29 in 200 μL PBS containing 3% hog gastric mucin-type III (Sigma Aldrich, St. Louis, MO, USA) and immediately imaged in both ventral and dorsal positions on the IVIS Lumina XRMS Series III system. Immediately thereafter, the mice in group 1 were orally administered with the vehicle, the mice in group 2 received oral NCL179 at 50 mg/kg, while the mice in group 3 received oral moxifloxacin at 6 mg/kg. The clinical conditions of all mice were closely monitored, and all mice were imaged at 2 h post-infection. In a preliminary investigation, a two-dose regime was designed for NCL179 such that the second dose was to be administered at 8 h post-infection with vehicle and moxifloxacin treatments given at 4 h, 8 h and 12 h post-infection. In a subsequent experiment, a four-dose regime was designed such that the 2nd, 3rd and 4th doses of the vehicle, NCL179 and moxifloxacin were given to the surviving mice in group 1, group 2 and group 3 at 4 h, 8 h and 12 h, respectively. At 4 h, 6h and 10 h post-infection, all surviving animals in each group were imaged. The mice were frequently monitored for signs of distress, and those that had become moribund or showed any evidence of distress were humanely killed by cervical dislocation. At 18 h, 24 h, 28 h, 36 h, 48 h and 72 h post-infection, the surviving mice were monitored and further subjected to bioluminescence imaging. At all imaging time points, signals were collected from a defined region of interest, and the total flux intensities (photons/s) were analyzed using Living Image Software 4.7.2. Differences in median survival times (time to moribund) for mice between groups were analyzed by the log-rank (Mantel-Cox) tests. Differences in luminescence signals between groups were compared by Mann-Whitney *U*-tests, two-tailed.

## 3. Results

### 3.1. NCL179 Shows In Vitro Activity against Gram-Positive Pathogens and also against Gram-Negative Pathogens in the Presence of Sub-Inhibitory Concentrations of Colistin

The antimicrobial activity of NCL179 was tested against GPB reference strains and clinical isolates, including 20 MRSA, 2 methicillin-sensitive *S. aureus* (ATCC 12600 [Xen29] and ATCC 29213), 20 MRSP, 20 VRE and 1 *E. faecalis* ATCC 29212. NCL179 showed MIC/MBCs of 1–2 µg/mL against MRSA and MRSP, MIC/MBCs 2–4 µg/mL against VRE compared to drug controls daptomycin (MIC/MBCs 0.5–2 µg/mL vs MRSA and VRE) and amikacin (MIC/MBCs 8–16 µg/mL vs MRSP) (Table 1). The MIC for NCL179 against Xen29 and *S. aureus* ATCC 29213 increased to 16 µg/mL in the presence of 10% FBS (Supplementary Materials Table S1).

The activity of the NCL179 + colistin combination was tested against GNB reference strains and clinical isolates (18 *A. baumannii*, 24 *E. coli*, 22 *K. pneumoniae* and 25 *P. aeruginosa*), and the results are presented in Table 2. NCL179 alone had no activity against GNB; therefore, its MIC was nominally taken as 256 µg/mL (the highest concentration tested) for FICI and DRI calculations. The MIC range of colistin alone against GNB was as follows: *A. baumannii* (0.5–2 µg/mL); colistin-resistant *A. baumannii* (64–128 µg/mL); *E. coli* (0.125–0.5 µg/mL); colistin-resistant *E. coli* (32 µg/mL); *K. pneumoniae* (0.25–2 µg/mL) and *P. aeruginosa* (0.25–2 µg/mL). However, for the NCL179 + colistin combination, the MIC range of NCL179 was 0.5–4 µg/mL against all tested GNB, whereas the MIC range of colistin was 0.008–0.5 µg/mL against *A. baumannii*, 0.5–1 µg/mL against colistin-resistant *A. baumannii*, 0.008–0.125 µg/mL against *E. coli*, 0.5 µg/mL against colistin-resistant *E. coli* and 0.015–0.5 µg/mL against *K. pneumoniae* and *P. aeruginosa.* The FICI of the NCL179 + colistin combination against all GNB showed synergistic activity, and all DRIs were beneficial (Table 2).

### 3.2. NCL179 Exhibits Time- and Concentration-Dependent Kill Kinetics of Bacterial Growth

**Figure 2:**
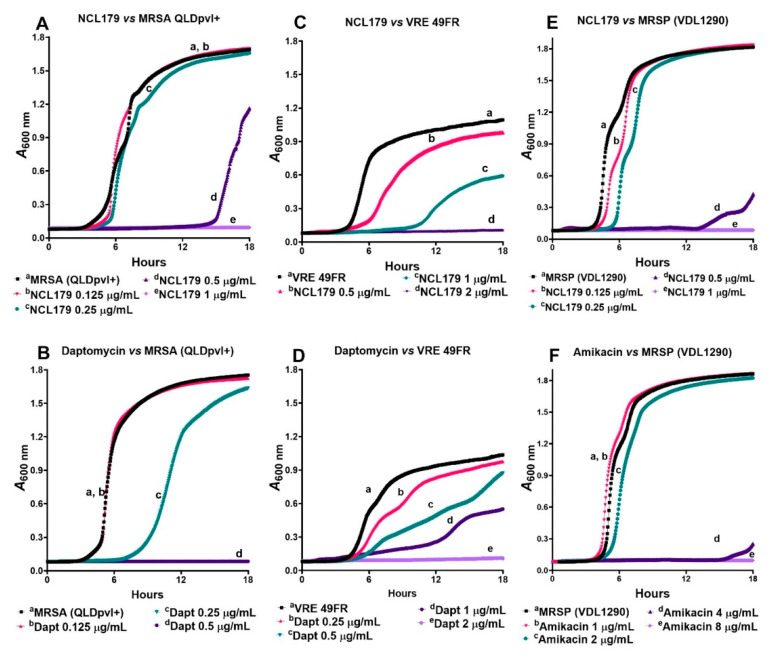
Time- and concentration-dependent kill kinetics of NCL179 against Gram-positive bacteria. (**A**), against MRSA QLDpvl+; (**C**), against VRE 49R; and (**E**), against MRSP (VDL1290) using daptomycin (**B**,**D**) and amikacin (**F**) as control drugs.

A time-kill kinetics assay was used to measure the time- and concentration-dependent activity of NCL179 against clinical MRSA isolate QLDpvl+, clinical MRSP isolate VDL1290 and clinical VRE isolate 49FR (Figure 2). Daptomycin was used as a drug control for the MRSA and VRE kill kinetics assay, while amikacin was used as a control drug for the MRSP kill kinetics assay. Our results show that NCL179 exhibited a time- and concentration-dependent killing of the bacteria as observed for the bactericidal control dugs.

**Figure 3:**
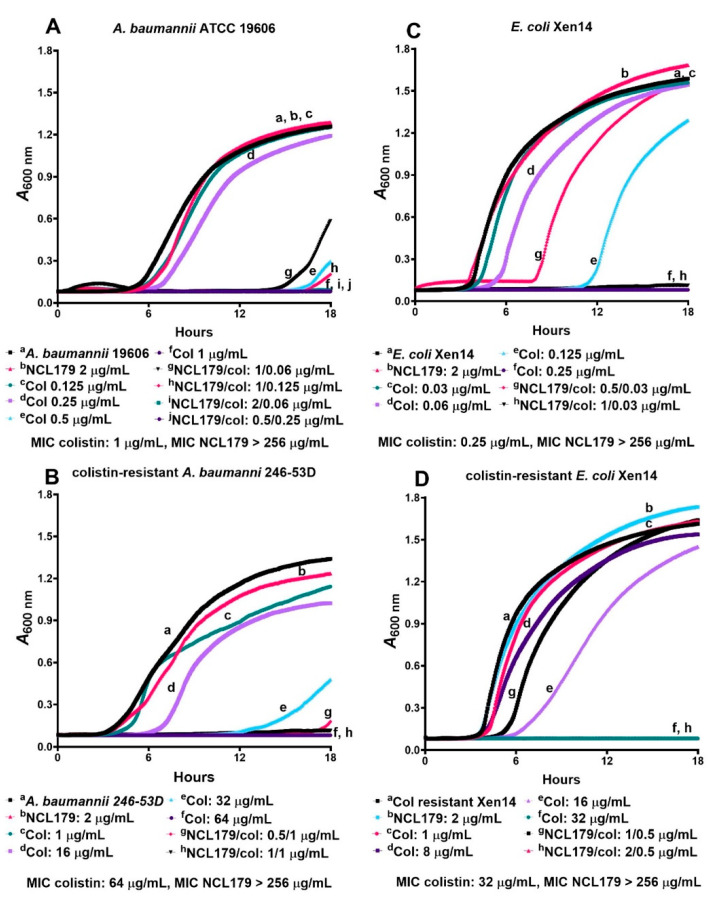
Time-and-concentration-dependent kill kinetics of NCL179 alone and in combination with colistin against Gram-negative bacteria. (**A**), NCL179 alone or in combination with colistin against *A. baumannii* ATCC 19606; (**B**), clinical colistin-resistant *A. baumannii* 246-53D; (**C**), *E. coli* Xen14; and (**D**), colistin-resistant *E. coli* Xen14. The concentrations of NCL179 and colistin for each combination were chosen based on data presented in Table 2. Assays were performed on a Cytation 5 Multi-Mode Reader (BioTek, Winooski, VT, USA) by optical density (*A*_600nm_) measurements; Col, colistin.

The time- and concentration-dependent activity of NCL179 + colistin combinations against GNB was also investigated in the kinetics assay using *A. baumannii* ATCC 19606, colistin-resistant clinical *A. baumannii* 246-53D, *E. coli* Xen14, colistin-resistant *E. coli* Xen14, *K. pneumoniae* ATCC 33495 and *P. aeruginosa* Xen41 (Figure 3 and Figure S2). The results indicate that, in each case, the NCL179 + colistin combination worked faster than colistin alone at the corresponding concentration. In addition, the NCL179 + colistin combination killed GNB, whereas cells treated with colistin alone at the corresponding concentrations still grew. As expected, NCL179 alone was not active against any of the GNB.

### 3.3. Combination of NCL179 and Colistin Shows Limited Cytotoxicity to Mammalian Cell Lines

**Figure 4:**
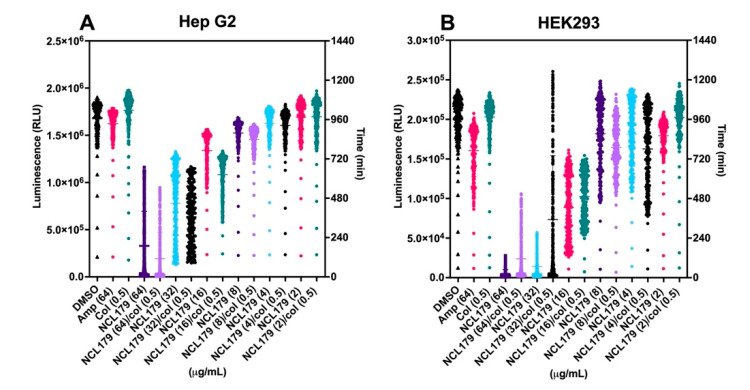
In vitro toxicity assessment of NCL179 alone and in combination with colistin. Real-time cell viability measurement for Hep G2 (**A**) and HEK293 (**B**) cells after treatment with different concentrations of NCL179 alone and in combination with 0.5 µg/mL of colistin. The cell viability was determined using the RealTime-Glo^TM^ MT Cell Viability Assay reagent (Promega, Madison, WI, USA) and measured every hour for 20 h at 37 °C in 5% CO_2_ on a Cytation 5 Cell Imaging Multi-Mode Reader (BioTek, BioTek, Winooski, VT, USA). Data are relative light units (RLU) for each treatment per time point; col, colistin; Amp, ampicillin.

The effects of various concentrations of NCL179 alone or in combination with 0.5 µg/mL colistin to Hep G2 (liver) and HEK293 (kidney) cell lines were examined in an in vitro cell toxicity assay. The results indicate an IC_50_ value of 16 µg/mL for NCL179 at the concentrations tested. In addition, the presence of colistin in the combination did not alter the IC_50_ value (Figure 4).

The hemolytic titer (HC_50_) for NCL179 was 32 μg/mL to human RBCs, whereas the HC_50_ for ampicillin was >128 μg/mL (the highest concentration used).

### 3.4. Agar Well Diffusion Shows NCL179 Remains Active in Formulations

**Figure 5:**
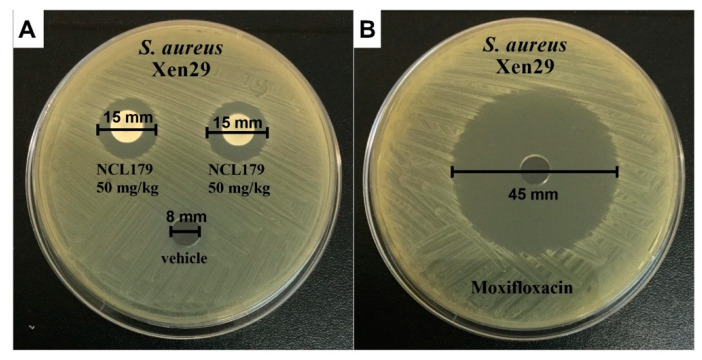
Selected well diffusion of NCL179 used in the efficacy trial. (**A**), to each well, 30 µL of either NCL179 formulation or vehicle only was added; (**B**), 30 µL of moxifloxacin (BovaVet, Caringbah, NSW, Australia). Concentrations of NCL179 in formulations were prepared as a 50 mg/mL solution to achieve 50 mg/kg in mice (30 g); moxifloxacin is a drug control and was prepared as a 6 mg/mL solution to achieve 6 mg/kg in mice (30 g); Xen29, *S. aureus* Xen29.

In order to ascertain that NCL179 retained its antimicrobial activity when formulated in the vehicle (20% [*v*/*v*] DMSO in PEG400), an agar diffusion test was performed. The formulations of NCL179 formed an inhibitory zone of 15 mm (Figure 5), indicating that NCL179 was released from the vehicle into the agar.

### 3.5. Oral Administration of NCL179 Shows Systemic Safety in Mice

**Figure 6:**
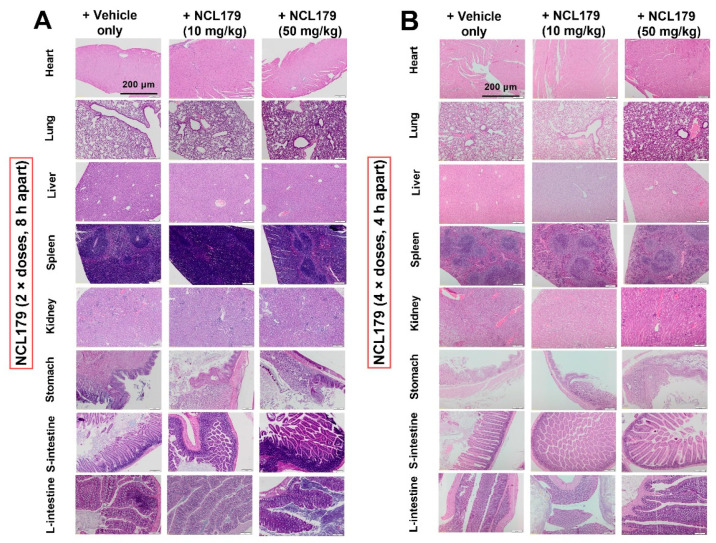
Representative histological images of heart, lung, liver, stomach, spleen, kidneys and small and large intestines from NCL179-treated and vehicle-treated mice. No histopathological changes were observed in mice treated orally with (**A**), 10 mg/kg, 50 mg/kg (2 × doses, 8 h apart), or (**B**), with 10 mg/kg, 50 mg/kg (4 × doses, 4 h apart) of NCL179 in comparison with vehicle. S-intestine, small intestine; L-intestine, large intestine. Scale bars: 200 µm.

There were no observable histopathological changes in the heart, lung, liver, spleen, stomach, kidneys and small and large intestines of mice orally treated with two doses of 10 mg/kg or 50 mg/kg NCL179 at 8 h apart (Figure 6A) or four doses of 10 mg/kg or 50 mg/kg NCL179 at 4 h apart (Figure 6B) in comparison with the vehicle at 72 h post-initial treatment.

### 3.6. Oral Treatment of Mice with NCL179 Reduces S. aureus Populations and Significantly Prolongs Survival Times

The potential of NCL179 as an oral therapeutic drug against systemic *S. aureus* infection was examined in an IP sepsis challenge mouse model using a well-characterized bioluminescent *S. aureus* strain (Xen29). Our preliminary investigations showed that an 8 h gap between the first and second NCL179 treatment was suboptimal as all mice treated with the first dose of NCL179 had succumbed to infection by the 8 h time point, similar to the vehicle-only treated group (Figure S3).

**Figure 7:**
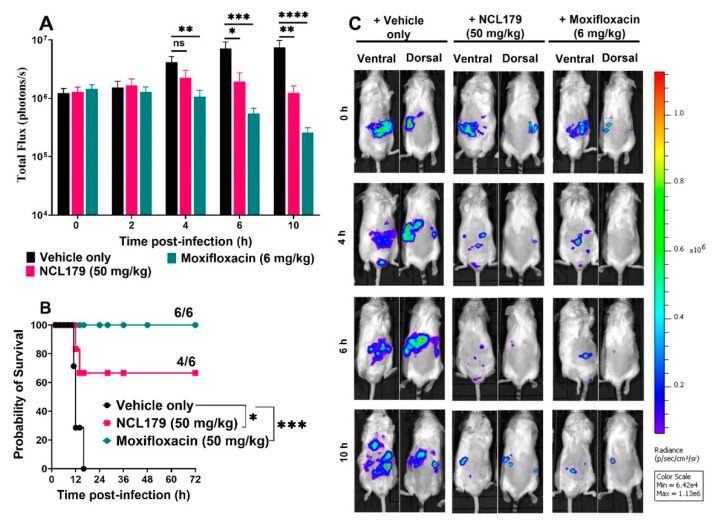
Oral efficacy of 4 doses of NCL179 given 4 h apart in a bioluminescent *S. aureus* Xen29 sepsis mouse model. (**A**), Comparison of luminescence signals between groups of CD1 mice (*n* = 6) challenged IP with Xen29 and treated with NCL179, moxifloxacin and vehicle at 0 h, 4 h, 8 h and 12 h post-infection. Mice were subjected to bioluminescence imaging on IVIS Lumina XRMS Series III system at the indicated times (ns, no significant; *, *p* < 0.05; **, *p* < 0.01; ***, *p* < 0.0002, ****, *p* <0.0001, Mann-Whitney *U*-test, two-tailed). (**B**), Survival analysis for mice treated with NCL179, moxifloxacin and vehicle (ns, no significant; *, *p* < 0.05; ***, *p* < 0.0002; Log-rank (Mantel-Cox test)). (**C**), Ventral and dorsal images of representative CD1 mice challenged with approx. 3 × 10^7^ CFU of bioluminescent *S. aureus* ATCC 12600 (Xen29). Moxifloxacin used as a control drug was prepared in almond oil at 6 mg/mL by BovaVet, Australia.

In a subsequent experiment, four oral doses of NCL179 at 4 h apart showed good efficacy (Figure 7). After the second NCL179 dose (at 4 h post-infection), there was a statistically significant reduction in *S. aureus* photon counts when measured at 6 h (*, *p* < 0.05, Mann-Whitney *U*-test, two-tailed). After a third NCL179 dose (at 8 h post-infection), *S. aureus* photon counts continued to reduce significantly when measured at 10 h (**, *p* < 0.01, Mann-Whitney *U*-test, two-tailed) (Figure 7A). The treatment of mice with four oral doses of NCL179 at 4 h apart also resulted in a significant increase in median survival time compared to the vehicle-only control (* *p* < 0.05; Mantel-Cox test; Figure 7B). The bacterial reduction caused by the different treatments could be clearly observed on bioluminescent images of the mice (Figure 7C).

## 4. Discussion

The increasing resistance of ESKAPE pathogens to commonly used antimicrobials in health care settings has led to a decrease in treatment options and continues to generate a high level of global public health concern [10]. Therefore, there is an urgent need to develop new broad-spectrum antimicrobials with activity against pan-resistant ESKAPE, particularly GNB pathogens, with a novel approach to overcoming the permeability barrier of the GNB outer membrane [12,25,35,36,37]. In this study, we demonstrate that a second robenidine analogue, NCL179, has the potential for further development as a future treatment for bacterial infections based on three major findings. Firstly, NCL179 demonstrated potent antimicrobial activity against a variety of GPB pathogens and also against GNB pathogens in the presence of a sub-inhibitory concentration of colistin. Secondly, NCL179 showed limited in vitro toxicity to mammalian cell lines, showed low hemolytic activity to human erythrocytes and demonstrated systemic safety in mice with no observed morphological effects on the major organs examined. Thirdly, the oral treatment of mice with NCL179 reduced *S. aureus* populations in vivo and significantly prolonged survival times.

In clinical settings, bacterial co-infection and superinfections are common, reducing treatment options [38], while the use of broad-spectrum antibiotics is advantageous for wider coverage of GNB and GPB pathogens [39]. In this study, we demonstrated that NCL179 showed bacterial activity against a wide range of clinical MDR GPB pathogens, including MRSA, MRSP and VRE. Importantly, a combination of NCL179 with sub-inhibitory concentrations of colistin resulted in a synergistic interaction against many GNB clinical isolates and reference strains, including *A. baumannii*, *E. coli*, *K. pneumoniae* and *P. aeruginosa* on the critical WHO priority list of bacteria for which new antibiotics are urgently needed [25,40].

The outer membrane is a distinctive feature of GNB that provides a barrier protecting GNB from exposure to unwanted chemical compounds, resulting in limited treatment options [12,41]. Among several proposed therapies for the further treatment of GNB infections [42,43], a combination of colistin with other antibiotics is considered a potential approach associated with overcoming the outer membrane permeability barrier, a broader antibacterial spectrum, synergistic effects and reduced risk for emerging resistance during therapy [20,44,45]. In addition, many studies have demonstrated that, in contrast to monotherapy, combination therapy may enhance antimicrobial effects and provide synergism [46,47,48,49,50]. In this study, the NCL179 + colistin combination showed synergistic activity against all tested GNB pathogens, including colistin-resistant *A. baumannii* and *E. coli* isolates, comparable to other synergy reports of combining colistin with other antibiotics [44,46,51]. In addition, it has been suggested that IV colistimethate sodium monotherapy may not be an optimal option if the MIC against GNB pathogens is higher than 1 µg/mL [46,52]. However, our results indicate that the MICs of colistin are from 0.008–1 µg/mL (representing a 4-128-fold dose reduction) in combination with NCL179. These results suggest that a combination of NCL179 with sub-inhibitory concentrations of colistin could potentially increase activity against colistin-resistant KAPE and other GNB. The combination could also reduce colistin toxicity levels, lower the likelihood of resistance development and decrease treatment time while increasing overall survival rate, as suggested by similar in vitro and in vivo models and analytical frameworks [53,54,55,56]. Therefore, the synergistic effect of NCL179 in combination with colistin is a promising development for a new chemical class scaffold to treat GNB infections.

The stringent criteria of a successful antibiotic require a balance between high in vivo efficacy and broad-spectrum antibacterial activity and safety to host targets (humans and animals) [57]. Here, we demonstrated the limited toxicity of NCL179 to mammalian cell lines, its low hemolytic activity, in vivo safety in mice, as well as the significant reduction in *S. aureus* populations in vivo and prolonged survival times of NCL179-treated mice. This opens the possibility of exploring NCL179 for human use after further testing in animal models of infection and chemical modification to identify more potent isosteres. Interestingly, the MIC of NC179 against *S. aureus* increases 16–fold in the presence of 10% FBS, suggesting a high level of protein binding, which could result in a low free NCL179 concentration in plasma, thereby limiting its systemic bioavailability. Nonetheless, this does not appear to have completely limited its effectiveness. Therefore, detailed pharmacokinetic studies of NCL179 in appropriate models need to be investigated, and the chemical modification of NCL179 is desirable to reduce plasma binding and potential toxicity and improve potency against the MDR pathogens. Together, this study demonstrates that NCL179 is a potential new chemical entity for further pharmaceutical development for the treatment of ESKAPE pathogens, either alone or in combination with existing antibiotic classes.

## 5. Conclusions

In this study, we demonstrated the in vitro antimicrobial activity of NCL179 against GPB and GNB in the presence of a sub-inhibitory concentration of colistin. The combination of NCL179 + colistin is expected to be another promising therapeutic option for the treatment of drug-resistant bacteria that pose the greatest health threats, particularly in the era of colistin-resistant GNB infections. By showing that the NCL179 + low dose colistin combination could safely and effectively overcome resistance to monotherapy with colistin, NCL179 provides a good example of the “5R” antimicrobial stewardship element, “Refinement” (5Rs: Responsibility, Reduction, Refinement, Replacement and Review) [58].

## Figures and Tables

**Table 1:** In vitro activity of NCL179 against Gram-positive ATCC strains and clinical isolates.

Strain/Isolate	^1^ MIC and ^2^ MBC Range (µg/mL)		
	NCL179	Daptomycin	Amikacin
^3^ MRSA (*n* = 20)	1–2	0.5–2	ND
^4^ MSSA (*n* = 2)	1	0.5	ND
^5^ MRSP (*n* = 20)	1–2	^7^ ND	8–16
^6^ VRE (*n* = 20)	2–4	0.5–2	ND

^1^ MIC, minimum inhibitory concentration; ^2^ MBC, minimum bactericidal concentration; ^3^ MRSA, methicillin-resistant *S. aureus*; ^4^ MSSA, methicillin-sensitive *S. aureus*; ^5^ MRSP, methicillin-resistant *S. pseudintermedius*; ^6^ VRE, vancomycin-resistant enterococci; and ^7^ ND, not determined. *E. faecalis* ATCC 29212 was used as a control Gram-positive strain, and it returned MIC and MBC values of 4 µg/mL for NCL179 and MIC and MBC values of 2 µg/mL for daptomycin.

**Table 2:** Synergistic activity of NCL179 in combination with colistin against reference strains and clinical isolates of Gram-negative bacteria.

Strain/Isolate	^1^ MIC Range (μg/mL)				^2^ FICI	^3^ DRI	
	Single Antibiotic		Combination				
	Colistin	NCL179	Colistin	NCL179		Colistin	NCL179
*A. baumannii* (*n* = 14)	0.5–2	>256	0.008–0.5	0.5–4	0.016–0.25 *	4–64	64–512
Colistin-resistant *A. baumannii* (*n* = 4)	64–128	>256	0.5–1	1–4	0.008 *	64-128	64–256
*E. coli* (*n* = 23)	0.125–0.5	>256	0.008–0.125	0.5–4	0.064–0.25 *	4–16	64–512
Colistin-resistant *E. coli* (*n* = 1)	32	>256	0.5	2	0.016 *	64	128
*K. pneumoniae* (*n* = 22)	0.25–2	>256	0.015–0.5	0.5–4	0.06–0.25 *	4–16	64–512
*P. aeruginosa* (*n* = 25)	0.25–2	>256	0.015–0.5	0.5–4	0.06–0.25 *	4–16	64–512

^1^ MIC, minimum inhibitory concentration; ^2^ FICI, fractional inhibitory concentration index: * synergistic, FICI ≤ 0.5; additive or partially synergistic, 0.5 < FICI ≤ 1; indifferent, 1 < FICI ≤ 4; and antagonistic, FICI > 4; ^3^ DRI, dose reduction index. Bioluminescent *S. aureus* Xen29 was used as control each time the MIC and checkerboard assays were performed; the MIC of NCL179 against *S. aureus* Xen29 in each of these assays was 1 μg/mL.

## Data Availability

The data presented in this study are available on request from the corresponding authors. The data are not publicly available due to privacy and access restrictions.

## Supplementary Materials

The following are available online at https://www.mdpi.com/article/10.3390/antibiotics11010065/s1, Figure S1: Representative checkerboard assay of NCL179 + colistin combination against *E. coli* ATCC 35218, Figure S2: Time- and concentration-dependent kill kinetics of NCL179 alone and in combination with colistin against Gram-negative bacteria; Figure S3: Oral efficacy of NCL179 (2 × doses, 8 h apart) in a bioluminescent *S. aureus* (Xen29) mouse sepsis model, Table S1: Effect of serum on in vitro activity of NCL179 against *S. aureus*.
