# Supplementary material for: Impact of a Novel Anticoccidial Analogue on Systemic Staphylococcus aureus Infection in a Bioluminescent Mouse Model
Source: Antibiotics (Basel). 2022 Jan 6;11(1):65. doi: 10.3390/antibiotics11010065 (PMC8773087; doi:10.3390/antibiotics11010065)
Supplement: Supplementary file 1 [file antibiotics-11-00065-s001.zip › antibiotics-1510893-supplementary.pdf]

Supplementary materials:

Table S1. Effect of serum on *in vitro* activity of NCL179 against *S. aureus*.

**Strain**	**[1] MIC (µg/mL) for:**			
	**NCL179**		**Daptomycin**	
	**[2] LB**	**LB + [3] 10% FBS**	**LB**	**LB + 10% FBS**
***S. aureus* Xen29**	1	16	0.5	0.5
***S. aureus* ATCC 29213**	1	16	0.5	0.5

[1] MIC, minimum inhibitory concentration; LB, Luria–Bertani broth; FBS, foetal bovine serum

	1	2	3	4	5	6	7	8	9	10	11	12	NCL179 (µg/mL)
**A**	1.7512	1.7298	0.0649	0.0564	0.0522	0.0577	0.0545	0.0602	0.0516	0.0626	0.0483	0.0508	**8**
**B**	1.7078	1.7108	1.6885	0.0524	0.0501	0.0459	0.0499	0.0523	0.0505	0.0534	0.0464	0.0468	**4**
**C**	1.6975	1.6986	1.6818	1.6416	0.0503	0.0403	0.0428	0.0429	0.0433	0.0431	0.0411	0.0405	**2**
**D**	1.6764	1.6804	1.6618	1.6124	1.6318	0.0398	0.0407	0.0415	0.0406	0.0418	0.0398	0.0403	**2**
**E**	1.665	1.6677	1.645	1.6302	1.6325	1.595	0.042	0.0424	0.0426	0.0414	0.0469	0.0429	**0.5**
**F**	1.6554	1.6418	1.6331	1.621	1.6303	1.5876	1.3484	0.0424	0.0418	0.0426	0.0411	0.0433	**0.25**
**G**	0.0414	1.5469	1.5311	1.5403	1.5766	1.5486	1.4634	0.0392	0.0393	0.0415	0.0404	0.0408	
**H**	0.0464	1.562	1.5558	1.5403	1.5645	1.5139	1.4283	0.0399	0.0407	0.043	0.0409	0.0424	
	**Growth (-)**	**Growth (+)**	**0.008**	**0.015**	**0.03**	**0.06**	**0.125**	**0.25**	**0.5**	**1**	**2**	**4**	**Colistin µg/mL**

Figure S1. Representative checkerboard assay of NCL179 + colistin combination against *E. coli* ATCC 35218. Values represent $A_{600\ nm}$ measurements. Colistin concentration ranged from 0.008 to 4 µg/mL (columns 3 to 12); NCL179 concentration ranged from 0.25 to 8 µg/mL (rows A to F); blue wells, NCL179 alone; green wells, colistin alone; red wells, bacterial growth control containing only *E. coli* and Luria–Bertani (LB) broth; orange wells, LB broth only (no growth control, also representing the $A_{600\ nm}$ value at the MIC of colistin alone or NCL179 + colistin combination)

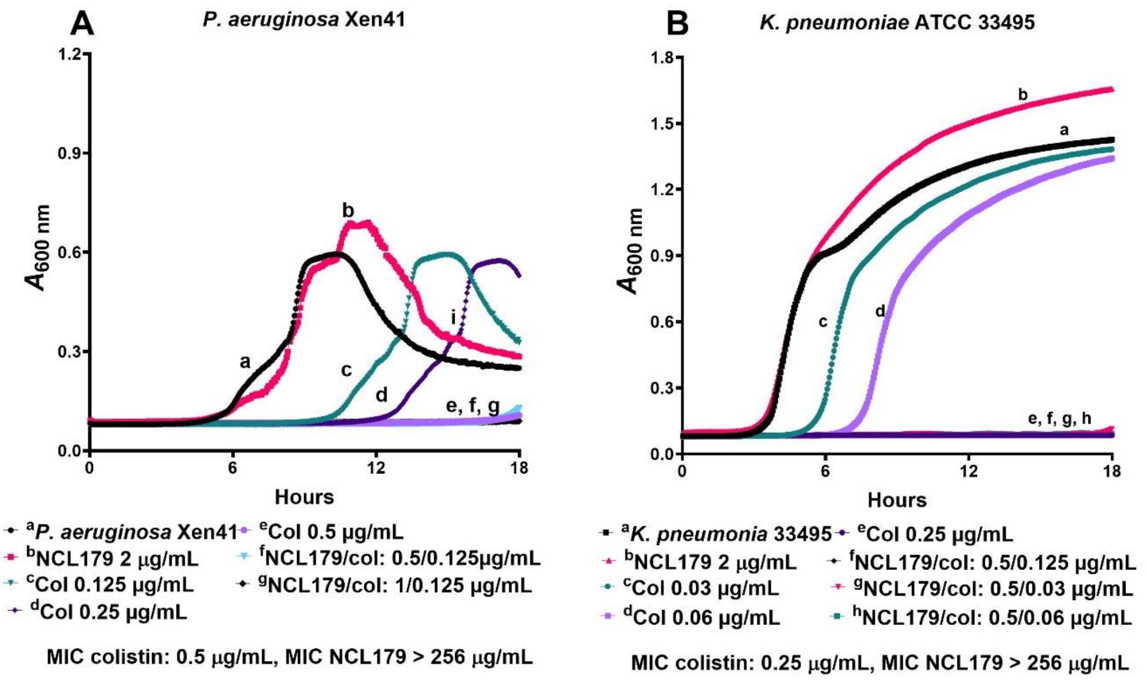

Figure S2. Time- and concentration-dependent kill kinetics of NCL179 alone and in combination with colistin against Gram-negative bacteria. (**A**), NCL179 alone or in combination with colistin against *P. aeruginosa* Xen41**,** and (**B**) *K. pneumoniae* ATCC 33495. Assays were performed on a Cytation 5 Multimode reader (BioTek) by optical density ($A_{600\ nm}$) measurements. Col, colistin

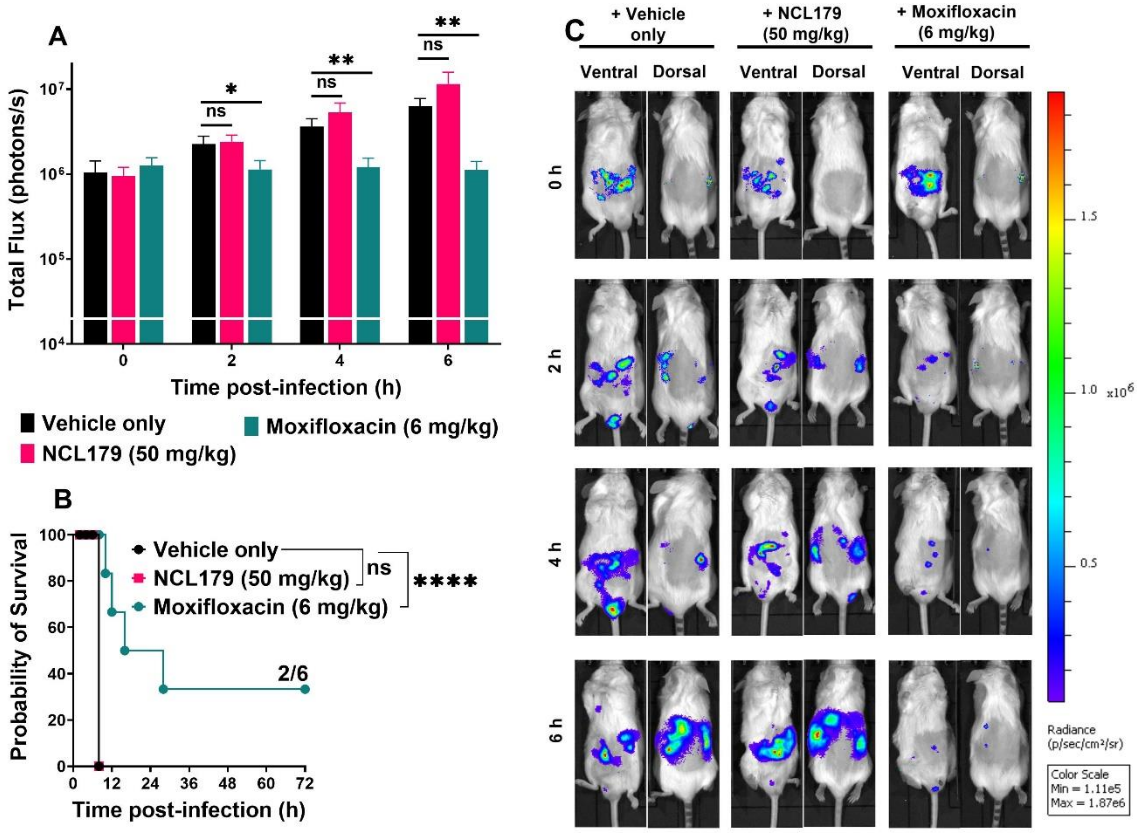

Figure S3. Oral efficacy of NCL179 (2 × doses, 8 h apart) in a bioluminescent *S. aureus* (Xen29) mouse sepsis model. (**A**) Comparison of luminescence signals between groups of CD1 mice (n = 6) challenged IP with Xen29 and treated with NCL179 and vehicle at 0 h post-infection; moxifloxacin at 0 h, 4 h, 8 h and 12 h post-infection. Mice were subjected to bioluminescence imaging on IVIS Lumina XRMS Series III system at the indicated times (ns, no significant; *, $p < 0.05$; **, $p < 0.01$; ***, $p < 0.0002$, ****, $p < 0.0001$, Mann-Whitney *U*-test, two-tailed); broken segment represents limit of detection (2×10^4 photons/s). (**B**) Survival analysis for mice treated with NCL179, moxifloxacin and vehicle (ns, no significant; *, $p < 0.05$; **, $p < 0.01$, ***, $p < 0.0002$, ****, $p < 0.0001$; Log-rank (Mantel-Cox test)). (**C**) Ventral and dorsal images of representative CD1 mice challenged with approx. 3×10^7 CFU of bioluminescent *S. aureus* ATCC 12600 (Xen29). Moxifloxacin (Avelox IV400 1.6 mg/mL; Bayer, Australia) was used as a control drug.
